# Statistical methods for analysis of single-cell RNA-sequencing data

**DOI:** 10.1016/j.mex.2021.101580

**Published:** 2021-11-17

**Authors:** Samarendra Das, Shesh N. Rai

**Affiliations:** a Division of Statistical Genetics, ICAR-Indian Agricultural Statistics Research Institute, PUSA, New Delhi 110012, India; b Biostatistics and Bioinformatics Facility, JG Brown Cancer Center, University of Louisville, Louisville, KY 40202, USA; c School of Interdisciplinary and Graduate Studies, University of Louisville, Louisville, KY 40292, USA; d Hepatobiology and Toxicology Center, University of Louisville, Louisville, KY 40202, USA; e Department of Bioinformatics and Biostatistics, University of Louisville, Louisville, KY 40202, USA; f Biostatistics and Informatics Facility, Center for Integrative Environmental Research Sciences, University of Louisville, Louisville, KY 40202, USA; g Christina Lee Brown Envirome Institute, University of Louisville, Louisville, KY 40202, USA

**Keywords:** Zero inflated negative binomial model, Molecular capture model, Observed UMI count, True UMI count, Mean, Zero Inflation, Overdispersion

## Abstract

Single-cell RNA-sequencing (scRNA-seq) is a recent high-throughput genomic technology used to study the expression dynamics of genes at single-cell level. Analyzing the scRNA-seq data in presence of biological confounding factors including dropout events is a challenging task. Thus, this article presents a novel statistical approach for various analyses of the scRNA-seq Unique Molecular Identifier (UMI) counts data. The various analyses include modeling and fitting of observed UMI data, cell type detection, estimation of cell capture rates, estimation of gene specific model parameters, estimation of the sample mean and sample variance of the genes, *etc*. Besides, the developed approach is able to perform differential expression, and other downstream analyses that consider the molecular capture process in scRNA-seq data modeling. Here, the external spike-ins data can also be used in the approach for better results. The unique feature of the method is that it considers the biological process that leads to severe dropout events in modeling the observed UMI counts of genes.

• The differential expression analysis of observed scRNA-seq UMI counts data is performed after adjustment for cell capture rates.

• The statistical approach performs downstream differential zero inflation analysis, classification of influential genes, and selection of top marker genes.

• Cell auxiliaries including cell clusters and other cell variables (*e.g.*, cell cycle, cell phase) are used to remove unwanted variation to perform statistical tests reliably.

## Graphical abstract

**Figure:**
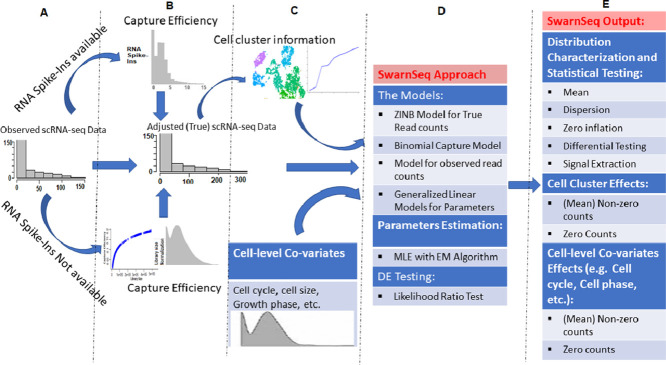

Specifications table

**Table:** 

Subject area	Statistics
More specific subject area	Statistical Genomics and Computational Biology
Method name	SwarnSeq
Name and reference of original method	Das, S. and Rai, S.N. (2021). SwarnSeq: An improved statistical approach for differential expression analysis of single-cell RNA-seq data. *Genomics*, **113**(**3**), 1308-1324.doi.org/10.1016/j.ygeno.2021.02.014
Resource availability	www.github/sam-uofl/SwarnSeq

## Data descriptions

We illustrated the performance of the methods on a publicly available single-cell RNA-seq (scRNA-seq) data. The full dataset was obtained from Yoruba (YRI) induced pluripotent stem cell (iPSC) lines, with three 96-well plates per individual [1]. We downloaded the Unique Molecular Identifier (UMI) counts, ERCC spike-in, and molecular concentration datasets from the github repository (https://github.com/jdblischak/singleCellSeq). We only used data of two individual cell lines NA19101 (288 cells) and NA19239 (288 cells) for further statistical analyses. The original UMI count data have expression values of genes/transcripts over 576 cells. To reduce the dimension of the data, we have removed the genes, which do not have non-zero expression values in at least five cells.

## Method details

***Notations:*** Let, $Y_{ijkl}$ be a random variable (rv) represents the observed (known) UMI counts in *i*^th^ *cell* (*i*= 1, 2,…, *I_k_*) for *j*^th^ gene (*j*= 1, 2,…, *J*) in *k*^th^ cell cluster (*k*= 1, 2,…, *K*) at *l*^th^ (*l=*1, 2,…, *L*) cell type/pseudo-time; $Z_{ijkl}$: rv represents unobserved/true (unknown) UMI counts in *i*^th^ cell for *j*^th^ gene in *k*^th^ cell cluster at *l*^th^ cell type/pseudo-time; *I_k:_* Number of cells present in *k*^th^ cell cluster; *I* $\left(=\sum_{k=1}^{K}I_{k}\right)$: total number of cells present in scRNA-seq data; *J*: total number of genes in the data; *K*: total number of cell clusters; *L*: number of cell types; $\mu_{ijkl}$ be the mean of non-zero counts in *i*^th^ cell for *j*^th^ gene in *k*^th^ cell cluster of *l*^th^ cell type; $\varphi_{ijkl}$ (=${\theta_{ijkl}}^{-1}$) and $\theta_{ijkl}$ be the dispersion and size parameters, respectively in i^th^ cell for *j*^th^ gene in *k*^th^ cell cluster of *l*^th^ cell type; $\pi_{ijkl}$ be the zero inflation probability in *i*^th^ cell for *j*^th^ gene in *k*^th^ cell cluster of *l*^th^ cell type.

## Traditional statistical models for fitting observed scRNA-seq data

### Negative binomial (NB) model

NB models are extensively used in modeling the read counts obtained from RNA-sequencing (RNA-seq) studies. The Probability Mass Function (PMF) of the NB distributional model is expressed in Eq. (1).

(1) $$f_{NB}\left(y\right)=P\left[Y_{ijkl}=y|\theta_{ijkl},\mu_{ijkl}\right]=\frac{G\left(y+\;\theta_{ijkl}\right)}{G\left(y+1\right)G\left(\theta_{ijkl}\right)}{\left(\frac{\theta_{ijkl}}{\theta_{ijkl}+\mu_{ijkl}}\right)}^{\theta_{ijkl}}{\left(\frac{\mu_{ijkl}}{\theta_{ijkl}+\mu_{ijkl}}\right)}^{y}\forall y=0,1,2,\;\ldots$$

where, $\mu_{ijkl}\geq 0;\;\theta_{ijkl}>0$ are the parameters of NB model, *G*(.): Gamma function. The NB distribution becomes Poisson, when $\theta_{ijkl}\to \;\infty$.

The mean and variance of the NB model is given in Eqs. (2) and (3), respectively.

(2) $$E\left(Y_{ijkl}\right)=\mu_{ijkl}$$

(3) $$Var\left(Y_{ij}\right)=\mu_{ijkl}+\frac{{\mu_{ijkl}}^{2}}{\theta_{ijkl}}=\mu_{ijkl}+{\mu_{ijkl}}^{2}\varphi_{ijkl}$$

### Zero inflated negative binomial (ZINB) model

The NB model implemented in bulk RNA-seq differential expression (DE) analytic tools including DESeq2, edgeR, baySeq, SAMSeq, *etc*., may not handle the excess overdispersion and zero inflation present in the single-cell UMI counts data [2,3]. Therefore, ZINB model is exclusively used for modeling/fitting of UMI count data obtained from single-cell studies [2], [3], [4], [5]. The ZINB model can be briefly described as follows:

The PMF of the ZINB distribution is given in Eq. (4).

(4) $$f_{ZINB}\left(y\right)=P\left[Y_{ijkl}=y|\pi_{ijkl},\theta_{ijkl},\mu_{ijkl}\right]=\pi_{ijkl}\delta_{0}\left(y\right)+\left(1-\pi_{ijkl}\right)f_{NB}\left(y\right)\;\forall \;y=0,\;1,\;2,\;\ldots$$

where, $f_{NB}\left(.\right):$ PMF of NB distribution (Eq. 1); $\delta_{0}\left(.\right):$ Dirac's delta function. Here, $\delta_{0}\left(.\right)$is used to model the excess zeros, and its PMF is equal to zero for every non-zero UMI counts and one for each zero-counts and can be expressed in Eq. (5).

(5) $$\delta_{0}\left(Y_{ijkl}=y\right)=\left\{ \begin{array}{cc} 1; & y=0\\ 0; & y\neq 0 \end{array}\right.$$

The PMF of the ZINB distribution, used to model the UMI counts from scRNA-seq studies, is given in Eq. (6).

(6) $$P\left[Y_{ijkl}=y\right]=\left\{ \begin{array}{cc} \pi_{ijkl}+\left(1-\pi_{ijkl}\right){\left(\frac{\theta_{ijkl}}{\theta_{ijkl}+\mu_{ijkl}}\right)}^{\theta_{ijkl}} & y=0\\ \left(1-\pi_{ijkl}\right)\frac{G\left(y+\;\theta_{ijkl}\right)}{G\left(y+1\right)G\left(\theta_{ijkl}\right)}{\left(\frac{\theta_{ijkl}}{\theta_{ijkl}+\mu_{ijkl}}\right)}^{\theta_{ijkl}}{\left(\frac{\mu_{ijkl}}{\theta_{ijkl}+\mu_{ijkl}}\right)}^{y}; & y>0 \end{array}\right.$$

If $\pi_{ijkl}=0$; $ZINB\left(\pi_{ijkl},\mu_{ijkl},\theta_{ijkl}\right)\to NB\left(\mu_{ijkl},\theta_{ijkl}\right)$

If $\theta_{ijkl}\to \infty \;\left(No\;dispersion\right);ZINB\left(\pi_{ijkl},\mu_{ijkl},\theta_{ijkl}\right)\;\to ZIP\left(\pi_{ijkl},\mu_{ijkl}\right)$ where, ZIP: Zero Inflated Poisson model.

## SwarnSeq model

In the existing single-cell data analytic tools including Seurat, DEsingle, Monocle, MAST, *etc*., the observed UMI counts are considered the realizations of true UMI counts. This assumption is not true, as different noises including biological sources, *e.g*., lower molecular capture, are mostly confounded with the observed UMI counts [2,4]. For instance, the recent single-cell sequencing protocols only capture the 1–10 % of the transcriptomics present in the cell [4,5]. Therefore, this property needs to be incorporated in modeling of the observed UMI count data. Here, we considered a simple Binomial cell capture model to model the observed UMI count data. However, other cellular capture model, *e.g*., Beta-Binomial, Poisson-NB models, Hypergeometric models, *etc*., can also be considered to represent biological dropout events in single-cell studies.

**Theorem**: Let, $\rho_{ijkl}$ be the rv represents the transcriptional capture rate of *i*^th^ cell for *j*^th^ gene in *k*^th^ cell cluster at *l*^th^ cell type/pseudo-time. If the true UMI counts, $Z_{ijkl}$, follow $ZINB\;(\pi_{ijkl},\mu_{ijkl},\theta_{ijkl})$ distribution, and $\rho_{ijkl}$ follows a binomial model with parameter $p_{ijkl}\left(0\leq p_{ijkl}\leq 1\right)$, then the observed UMI counts, $Y_{ijkl}$, will also follow ZINB distribution with parameters $(\pi_{ijkl},\mu_{ijkl}p_{ijkl},\theta_{ijkl}).$

**Proof:** Given that, $Z_{ijkl}∼\;ZINB\left(\pi_{ijkl},\mu_{ijkl},\theta_{ijkl}\right)\text{and}\;\rho_{ijkl}=\left(Y_{ijkl}|Z_{ijkl}=z\right)∼\;B\left(z,p_{ijkl}\right)$

Now, the PMF of $Z_{ijkl}$ is given in Eq. (4) and the PMF of $\rho_{ijkl}$ can be expressed in Eq. (7).

(7) $$P\left[Y_{ijkl}=y|Z_{ijkl}=z\right]=\left(\begin{array}{c} z\\ y \end{array}\right){p_{ijkl}}^{y}{\left(1-p_{ijkl}\right)}^{z-y}$$

The joint probability distribution of the observed and true UMI counts, $Y_{\mathrm{ijkl}}\;\text{and}\;Z_{\mathrm{ijkl}}$, can be written as:

(8) $$P\left[Y_{ijkl}=y,\;Z_{ijkl}=z|\pi_{ijkl},\mu_{ijkl},\theta_{ijkl},p_{ijkl}\right]=P\left[Y_{ijkl}=y|Z_{ijkl}=\;z,\;p_{ijkl}\right]P\left[Z_{ijkl}=z|\pi_{ijkl},\mu_{ijkl},\theta_{ijkl}\right]$$

Now, the marginal probability distribution of $Y_{ijkl}$ can be obtained as:

(9) $$P\left[Y_{ijkl}=y|\pi_{ijkl},\mu_{ijkl},\theta_{ijkl},p_{ijkl}\right]=\sum_{z}P\left[Y_{ijkl}=y|Z_{ijkl}=z,\;p_{ijkl}\right]P\left[Z_{ijkl}=z|\pi_{ijkl},\mu_{ijkl},\theta_{ijkl}\right]$$

*Case-1: when observed UMI count is zero* (*i.e.,* $Y_{ijkl}=0$)

(10) $$\begin{array}{ccc} & & P\left[Y_{ijkl}=0|\pi_{ijkl},\mu_{ijkl},\theta_{ijkl},p_{ijkl}\right]\\ & & =\pi_{ijkl}+\left(1-\pi_{ijkl}\right){\left(\frac{\theta_{ijkl}}{\theta_{ijkl}+{\mu^{\prime }}_{ijkl}}\right)}^{\theta_{ijkl}}\left(\mu_{ijkl}p_{ijkl}={\mu^{\prime }}_{ijkl}\;\left(say\right)\right) \end{array}$$

*Case-2: when observed UMI count is non-zero* (*i.e.,* $Y_{ijkl}\left(>0\right)=t=1,\;2,\;3,\ldots$)

(11) $$\begin{array}{ccc} & & P\left[Y_{ijkl}=t|\pi_{ijkl},\mu_{ijkl},\theta_{ijkl},p_{ijkl}\right]\\ & & =\left(1-\pi_{ijkl}\right)\frac{G\left(t+\;\theta_{ijkl}\right)}{G\left(t+1\right)G\left(\theta_{ijkl}\right)}{\left(\frac{\theta_{ijkl}}{\theta_{ijkl}+{\mu^{\prime }}_{ijkl}}\right)}^{\theta_{ijkl}}{\left(\frac{{\mu^{\prime }}_{ijkl}}{\theta_{ijkl}+{\mu^{\prime }}_{ijkl}}\right)}^{t} \end{array}$$

Now, Eqs. (10) and (11) are in the form of Eq. (4), which indicates the distribution of the observed UMI counts, $Y_{ijkl}$, is also from $ZINB(\pi_{ijkl},{\mu^{\prime }}_{ijkl},\theta_{ijkl}).$ The detailed proof of this theorem can be found at [2].

**Corollary 1**: When $p_{ijkl}=1$ (*i.e.*, under full capture rates), this means that all the transcriptomic material present in the cell is fully captured during the sequencing process, this is called as perfect deep sequencing. Under such scenarios, the distributions of the observed and true UMI counts remain same, *i.e.*, a ZINB model. Mathematically,

(12) $$\mathrm{ZINB}\left(\pi_{\mathrm{ijkl}},{\mu^{\prime }}_{\mathrm{ijkl}},\theta_{\mathrm{ijkl}}\right)\overset{d}{\to }\;\mathrm{ZINB}\left(\pi_{\mathrm{ijkl}},\mu_{\mathrm{ijkl}},\theta_{\mathrm{ijkl}}\right)$$

Here, the genes in a cell will have zero counts which are not truly expressed (*i.e.,* biological zeros) and the single-cell experiment will be free from dropout events. However, such a scenario is a dream in real experimental single-cell studies. In other words, the real limits of $p_{ijkl}$ is $0<p_{ijkl}<1$.

**Corollary 2**: In case $p_{ijkl}<1$, *i.e.*, in real experimental case the transcriptomic materials present in cells is not fully captured, but only certain fraction is captured [9]. Then, zero counts in the single-cell expression data are the mixture of dropout/false zeros and true zeros. Further, mean of the observed non-zero UMI counts depend on the cell capture rate parameter, while the zero inflation and overdispersion parameters are independent of the cell capture rates. Here, it is worthy to note that ${\hat{\pi }}_{ijkl}$ from observed data can be used to estimate the proportions of true zeros, as $\pi_{ijkl}$ remains unaffected by the capture rate parameter.

(13) $$\text{True}\;\text{UMI}\;\text{counts}:Z_{ijkl}\;∼\;ZINB\left(\pi_{ijkl},\mu_{ijkl},\theta_{ijkl}\right)$$

(14) $$\text{Observed}\;\text{UMI}\;\text{counts}:Y_{ijkl}\;∼\;ZINB\left(\pi_{ijkl},{\mu^{\prime }}_{ijkl},\theta_{ijkl}\right),\mu_{ijkl}^{\prime }=\mu_{ijkl}p_{ijkl}$$

In single-cell experiments, the observed UMI counts are noisy reflection of the true expression of genes due to lower cellular transcriptional capturing Eqs. (13), ((14)). In other words, distributions of the observed UMI counts of genes are the joint distributions of gene's true expression and transcriptional (cell) capture rate. The relation between the true and observed means of non-zero counts of genes is $\mu_{ijkl}>\mu_{ijkl}^{\prime }\;$. This means, the distribution of observed UMI counts will shift more towards zero, if the cellular capture rate is decreased. In other words, weightage of the Dirac's delta function will be more in the mixture distribution (Eq. (4)) compared to be NB part.

### Expected value and variance of the observed UMI counts in SwarnSeq model

The expected value and variance of the observed UMI counts of genes, $Y_{ijkl},$ in the SwarnSeq model can be expressed in Eq. (15).

(15) $$E\left(Y_{ijkl}\right)=\left(1-\pi_{ijkl}\right)\mu_{ijkl}p_{ijkl}$$

(16) $$V\left(Y_{ijkl}\right)=\left(1-\pi_{ijkl}\right)\mu_{ijkl}p_{ijkl}\left(1+\pi_{ijkl}\mu_{ijkl}p_{ijkl}+\mu_{ijkl}p_{ijkl}\varphi_{ijkl}\right)$$

In the SwarnSeq method, expected value of the observed UMI counts of genes depends on the zero inflation, mean of non-zero counts, and cell capture rate parameter. While the variance of the observed UMI counts are the functions of the zero inflation, mean of non-zero counts, overdispersion, and cell capture rate parameters. Further, the relation between the variance and expected value of the observed UMI counts of genes can be shown in Eq. (17). Alternatively, variance of the observed UMI counts of a gene is the function of its expected values (Eq. (17)) (*i.e.*, case of overdispersion).

(17) $$V\left(Y_{ijkl}\right)=E\left(Y_{ijkl}\right)\left\{1+\mu_{ijkl}p_{ijkl}\left(\pi_{ijkl}+\varphi_{ijkl}\right)\right\}$$

### Distributions of sample mean and sample variance of observed counts of genes

Usually, population parameters of the genes including population mean and variance are unknown, and they are estimated from experimentally observed sample UMI count data. Hence, it is important to obtain the sampling distribution of sample means and variances of the genes in a single-cell experimental study. The sample mean and variance of the observed UMI counts for *j*^th^ gene can be expressed in Eqs. (18), and (19), respectively. Here, for simplicity, we omitted the subscript denoting cell type.

(18) $${\bar{y}}_{j}=\frac{1}{K}\sum_{k=1}^{K}\frac{1}{I_{k}}\sum_{i=1}^{I_{k}}Y_{ijk}$$

(19) $$s_{j}^{2}=\frac{1}{K}\sum_{k=1}^{K}\frac{1}{\left(I_{k}-1\right)}\sum_{i=1}^{I_{k}}{\left(Y_{ijk}-{\bar{y}}_{j}\right)}^{2}$$

The expected values of the gene sample mean, and sample variance of the observed UMI counts can be derived under certain statistical assumptions. In other words, we assume that the observed count data are drawn from the ZINB population model, as given in Eq. (4), and the transcriptional capture efficiencies of the genes remain same. Further, the model parameters for the genes remain same over the cells in different cell clusters, *i.e.,* $\mu_{1j1}=\cdots =\mu_{I_{1}j1}=\cdots =\;\mu_{I_{K}\mathrm{jK}}=\mu_{j}$; $\pi_{1j1}=\cdots =\pi_{I_{1}j1}\;\cdots =\mu_{I_{K}\mathrm{jK}}=\pi_{j}$; $\theta_{1j1}=\ldots =\theta_{I_{1}j1}\ldots =\theta_{I_{K}jK}=\theta_{j};$

(20) $$p_{i1k}=p_{i2k}=\ldots =p_{iJk}=p_{ik}$$

Now, the theoretical expression of expected value of the sample mean for *j*^th^ gene can be derived as:

(21) $$\begin{array}{ccc} E\left({\bar{y}}_{j}\right) & = & \frac{1}{K}\sum_{k=1}^{K}\frac{1}{I_{k}}\sum_{i=1}^{I_{k}}E\left(Y_{ijk}\right)=\frac{1}{K}\sum_{k=1}^{K}\frac{1}{I_{k}}\sum_{i=1}^{I_{k}}E\left\{E\left(Y_{ijk}|Z_{ijk}\right)\right\}\\ & & =\frac{1}{K}\sum_{k=1}^{K}\frac{1}{I_{k}}\sum_{i=1}^{I_{k}}\left(1-\pi_{ijkl}\right)\left(\mu_{ijk}p_{ijk}\right) \end{array}$$

Under the assumption of Eq. (20), the expected value of sample mean for *j*^th^ gene (Eq. (21)) can be obtained, as shown in Eq. (22).

(22) $$E\left({\bar{y}}_{j}\right)=\frac{1}{K}\sum_{k=1}^{K}\frac{1}{I_{k}}\sum_{i=1}^{I_{k}}\left(1-\pi_{j}\right)\mu_{j}p_{ik}=\mu_{j}\left(1-\pi_{j}\right)\frac{1}{K}\sum_{k=1}^{K}\frac{1}{I_{k}}\sum_{i=1}^{I_{k}}p_{ik}=\mu_{j}\left(1-\pi_{j}\right){\bar{p}}_{..}$$

The variance of the observed UMI data, $V(Y_{ijk})$, (Eq. (16)) under the assumption of Eq. (20), becomes:

(23) $$V\left(Y_{ijk}\right)=\left(1-\pi_{j}\right)\mu_{j}p_{ik}\left(1+\pi_{j}\mu_{j}p_{ik}+\mu_{j}p_{ik}\varphi_{j}\right)$$

Now, the variance of sample mean (Eq. (18)) can be obtained as shown in Eq. (24) under the assumption of Eq. (20).

(24) $$V\left({\bar{y}}_{j}\right)=E\left({\bar{y}}_{j}^{2}\right)-{\left\{E\left({\bar{y}}_{j}\right)\right\}}^{2}=\frac{\mu_{j}\left(1-\pi_{j}\right)}{I}\left\{2\bar{p}+\mu_{j}\varphi_{j}\bar{p_{\cdot \cdot }^{2}}\right\}+{\left(1-\pi_{j}\right)}^{2}{\mu_{j}}^{2}\text{var}\left(p_{ik}\right)$$

Let, $s_{j}^{2}$ be the sample variance of *j*^th^ gene, expressed in Eq. (19). Then its expected value can be derived as follows.

(25) $$E\left(s_{j}^{2}\right)=\frac{1}{K}\sum_{k=1}^{K}\frac{1}{\left(I_{k}-1\right)}\sum_{i=1}^{I_{k}}\left\{V\left(Y_{ijk}\right)+E{\left(Y_{ijk}\right)}^{2}\right\}-\frac{1}{K\left(K-1\right)}\sum_{k\neq k^{\prime }=1}^{K}\frac{1}{I_{k}\left(I_{k}-1\right)}\sum_{i\neq i^{\prime }=1}^{I_{k}}E\left(Y_{ijk}\right)E\left(Y_{i^{\prime }jk^{\prime }}\right)=\mu_{j}{\bar{p}}_{..}+{\mu_{j}}^{2}\varphi_{j}\bar{p_{..}^{2}}+{\mu_{j}}^{2}var\left(p_{ik}\right)$$

where, ${\bar{p}}_{..}=\frac{1}{K}\sum_{k=1}^{K}\frac{1}{I_{k}}\sum_{i=1}^{I_{k}}p_{ik}$, ${\bar{p^{2}}}_{..}=\frac{1}{K}\sum_{k=1}^{K}\frac{1}{I_{k}}\sum_{i=1}^{I_{k}}p_{ik}^{2}$ and $var(p_{ik})$is the variance of $p_{ik}.$ *I* is the total number of cells, *i.e.*, $I=\sum_{k=1}^{K}I_{k}$.

### Estimation of SwarnSeq model parameters

We have shown that the distribution of sample means and variances of genes in experimental single-cell studies depends on gene specific model parameters, which are unknown. So, it is necessary to estimate them to get the exact distribution of gene specific sample statistic(s) and performing other analyses including DE analysis. Here, the parameters of the SwarnSeq model, given in Eqs. (10) and (11), were estimated from the observed UMI count data (adjusted for cell capture rates) under a Generalized Linear Model (GLM) framework. We have shown that the observed UMI counts for *j*^th^ gene, $Y_{ijk}$, as a ZINB *rv* with parameters: $\mu_{j}^{\prime }=\left(\mu_{1j1}^{\prime },\ldots ,\mu_{I_{1}j1}^{\prime },\;\ldots \mu_{I_{2}j2}^{\prime },\ldots ,\;\mu_{I_{K}jK}^{\prime }\right)$; $\pi_{j}=\left(\pi_{1j1},\cdots ,\pi_{I_{1}j1},\;\cdots ,\pi_{I_{2}j2},\cdots ,\mu_{I_{K}\mathrm{jK}}\right)$; $\theta_{k}=\left(\theta_{1j1},\ldots ,\theta_{I_{1}j1},\;\ldots ,\theta_{I_{2}j2},\ldots ,\theta_{I_{K}jK}\right)$ and further the following GLMs Eqs. (26)–((28)) are considered to model these parameters in the presence of cell-level co-variates and cell cluster data.

(26) $$\alpha_{j}=\log\mu_{j}^{\prime }=X\gamma_{j}+Rw_{j}+Cs_{j}+O_{\mu }$$

(27) $$\tau_{j}=\mathrm{logit}\pi_{j}=X\beta_{j}+Ru_{j}+Cv_{j}+O_{\pi }$$

(28) $$\omega_{j}=\log\theta_{j}$$

where, $logit\left(\pi_{j}\right)=log\left(\frac{\pi_{j}}{1-\pi_{j}}\right)$; $\alpha_{j}$**,** $\tau_{j}$ and $\omega_{j}$**:** *I* × 1 vector of parameters for *j*^th^ gene; ***X*:** *I* × *L* design matrix providing group information (first column consists of 1’s to include intercept term); *L*: number of cellular groups/types (cell clusters are divided into *L* cell groups, if cell group is unknown); ***R***: *I* × *K* design matrix providing cell cluster information; ***C***: *I* × *C* design matrix providing other cell level auxiliary information; $\gamma_{j}$ and $\beta_{j}$**:** *L* × *1* vectors of cellular groups effects for *j*^th^ gene; $w_{j}$ and $u_{j}$: *K* × *1* vectors of cell cluster effects for *j*^th^ gene; $s_{j}$ and $v_{j}$: *C* × *1* vectors of effects for other cell level co-variates including cell cycle, cell phase, *etc.* for the *j*^th^ gene; *C*: Levels of cell level auxiliaries. $O_{\mu },O_{\pi }$: offsets for $\mu_{j}^{\prime }$ and $\pi_{j}$ respectively.

### Expectation maximization (EM) algorithm

The parameters in Eqs. (26)–(28) for *j*^th^ gene, *i.e.,* $\Omega_{j}=\left\{\alpha_{j},\tau_{j},\omega_{j}\right\}$ can be estimated by using the Maximum Likelihood Estimation (MLE) Method. It is very difficult to obtain closed form solutions for the resulting log-likelihood function, given in Eq. (29). So, we developed an EM algorithm to estimate the SwarnSeq model parameters. For simplicity, we omit the subscripts for cellular type/pseudo-time in the notations. For the EM algorithm, we recast our estimation procedure into a missing data problem through introducing a latent rv, $V_{ijk}$, as defined in Eq. (30). Further, the incomplete data likelihood function for *j*^th^ gene can be expressed as:

(29) $$L\left(\Omega_{j};Y_{ijk}=y_{ijk}\right)=\prod_{k=1}^{K}\prod_{i=1}^{I_{k}}\left\{\pi_{ijk}\delta_{0}\left(y_{ijk}\right)+\left(1-\pi_{ijk}\right)f_{NB}\left(y_{ijk}\right)\right\}$$

(30) $$V_{ijk}=\left\{ \begin{array}{cc} 1\;\text{if}\;Y_{ijk} & \text{comes}\;\text{from}\;\text{the}\;\text{zero}\;\text{componet}\\ 0\;\text{if}\;Y_{ijk} & \text{comes}\;\text{from}\;\text{the}\;\text{count}\;\text{component} \end{array}\right.$$

Now, the joint likelihood function for complete data (in presence of latent variable), *i.e.,*$\left(Y_{ijk},V_{ijk}\right)$ can be expressed in Eq. (31), as:

(31) $$\begin{array}{ccc} & & L\left(\Omega_{j};Y_{\mathrm{ijk}},V_{\mathrm{ijk}}\right)\\ & & =\left[{\left\{\pi_{\mathrm{ijk}}+\left(1-\pi_{\mathrm{ijk}}\right){\left(\frac{\theta_{\mathrm{ijk}}}{\theta_{\mathrm{ijk}}+{\mu^{\prime }}_{\mathrm{ijk}}}\right)}^{\theta_{\mathrm{ijk}}}\right\}}^{V_{\mathrm{ijk}}}{\left\{\left(1-\pi_{\mathrm{ijk}}\right)\frac{G\left(z+\;\theta_{\mathrm{ijk}}\right)}{G\left(z+1\right)G\left(\theta_{\mathrm{ijk}}\right)}{\left(\frac{\theta_{\mathrm{ijk}}}{\theta_{\mathrm{ijk}}+{\mu^{\prime }}_{\mathrm{ijk}}}\right)}^{\theta_{\mathrm{ijk}}}{\left(\frac{{\mu^{\prime }}_{\mathrm{ijk}}}{\theta_{\mathrm{ijk}}+{\mu^{\prime }}_{\mathrm{ijk}}}\right)}^{y_{\mathrm{ijk}}}\right\}}^{1-V_{\mathrm{ijk}}}\right] \end{array}$$

Then, the log-likelihood function in Eq. (31) becomes:

(32) $$\begin{array}{ccc} & & l\left(\Omega_{j};Y_{ijk},V_{ijk}\right)\\ & & =\sum_{k=1}^{K}\sum_{i=1}^{I_{k}}V_{ijk}log\left\{\pi_{ijk}+\left(1-\pi_{ijk}\right){\left(\frac{\theta_{ijk}}{\theta_{ijk}+{\mu^{\prime }}_{ijk}}\right)}^{\theta_{ijk}}\right\}\\ & & +\;\sum_{k=1}^{K}\sum_{i=1}^{I_{k}}\left(1-V_{ijk}\right)log\left\{\left(1-\pi_{ijk}\right)\frac{G\left(z+\;\theta_{ijk}\right)}{G\left(z+1\right)G\left(\theta_{ijk}\right)}{\left(\frac{\theta_{ijk}}{\theta_{ijk}+{\mu^{\prime }}_{ijk}}\right)}^{\theta_{ijk}}{\left(\frac{{\mu^{\prime }}_{ijk}}{\theta_{ijk}+{\mu^{\prime }}_{ijk}}\right)}^{y_{ijk}}\right\}\\ & & =l_{1}\left(\Omega_{j};V_{ijk}\right)+l_{2}\left(\Omega_{j};Y_{ijk},V_{ijk}\right) \end{array}$$

where, $l_{1}\left(.\right)$: log-likelihood due to the zero-component of the model and $l_{2}\left(.\right)$: log-likelihood due to the count-component of the model. Further, the expected value of the log-likelihood function (Eq. (32)) can be obtained as:

(33) $$\begin{array}{ccc} Q & = & E\left[l\left(\Omega_{j};Y_{\mathrm{ijk}}=y,V_{\mathrm{ijk}}\right)\right]=\sum_{k=1}^{K}\sum_{i=1}^{I_{k}}E\left(V_{\mathrm{ijk}}|Y_{\mathrm{ijk}},\Omega_{j}\right)\log\left\{\pi_{\mathrm{ijk}}+\left(1-\pi_{\mathrm{ijk}}\right){\left(\frac{\theta_{\mathrm{ijk}}}{\theta_{\mathrm{ijk}}+{\mu^{\prime }}_{\mathrm{ijk}}}\right)}^{\theta_{\mathrm{ijk}}}\right\}\\ & & +\;\sum_{k=1}^{K}\sum_{i=1}^{I_{k}}\left(w_{\mathrm{ijk}}\right)\log\left\{\left(1-\pi_{\mathrm{ijk}}\right)\frac{G\left(y+\;\theta_{\mathrm{ijk}}\right)}{G\left(y+1\right)G\left(\theta_{\mathrm{ijk}}\right)}{\left(\frac{\theta_{\mathrm{ijk}}}{\theta_{\mathrm{ijk}}+{\mu^{\prime }}_{\mathrm{ijk}}}\right)}^{\theta_{\mathrm{ijk}}}{\left(\frac{{\mu^{\prime }}_{\mathrm{ijk}}}{\theta_{\mathrm{ijk}}+{\mu^{\prime }}_{\mathrm{ijk}}}\right)}^{y_{\mathrm{ijk}}}\right\} \end{array}$$

The conditional expectations in Eq. (33) can be given as:

(34) $$E\left(V_{ijk}|Y_{ijk}=y_{ijk},\Omega_{j}\right)=P\left[V_{ijk}=1|Y_{ijk},\Omega_{j}\right]=\frac{\pi_{ijk}+\left(1-\pi_{ijk}\right){\left(\frac{\theta_{ijk}}{\theta_{ijkl}+{\mu^{\prime }}_{ijk}}\right)}^{\theta_{ijk}}}{\pi_{ijk}\delta_{0}\left(y_{ijk}\right)+\left(1-\pi_{ijk}\right)f_{NB}\left(y_{ijk};{\mu^{\prime }}_{ijk},\;\theta_{ijk}\right)}$$

The posterior probabilities or the conditional weights in Eqn 33 for observations originate from the count component of the model and can be given as:

(35) $$w_{ijk}=1-E\left(V_{ijk}|Y_{ijk},\Omega_{j}\right)=P\left[V_{ijk}=0|Y_{ijk},\Omega_{j}\right]=\frac{\left(1-\pi_{ijk}\right)f_{NB}\left(y_{ijk};{\mu^{\prime }}_{ijk},\;\theta_{ijk}\right)}{\pi_{ijk}\delta_{0}\left(y_{ijk}\right)+\left(1-\pi_{ijk}\right)f_{NB}\left(y_{ijk};{\mu^{\prime }}_{ijk},\;\theta_{ijk}\right)}$$

where, $f_{NB}\left(.\right)$ is the PMF of NB distribution given in Eq. (1).

***E-step***: The E-step in the EM algorithm involves in evaluating the expected value of the log-likelihood of the complete data (Eq. (33)), given the observed data with current estimates of the parameters. In this approach, for each gene, given the observed data and the current estimate of the ZINB parameters, the expected value of the log-likelihood is calculated. Let, ${{\hat{\Omega }}_{j}}^{c}=\left\{{{\hat{\alpha }}_{j}}^{c},{{\hat{\tau }}_{j}}^{c},{{\hat{\varphi }}_{j}}^{c}\right\}$ be the given current estimate of the parameters, then the expected value of log likelihood (Eq. (33)) at step (c + 1), *i.e.,* $Q^{c+1}$ is calculated. The conditional expectation at *c*^*th*^ step, *i.e.,* $E(V_{ijk}|Y_{ijk},{{\hat{\Omega }}_{j}}^{c})$(Eqn 33)) can be estimated using Eq. (36).

(36) $$E\left(V_{\mathrm{ijk}}|Y_{\mathrm{ijk}},{{\hat{\Omega }}_{j}}^{c}\right)=\frac{{\hat{\pi }}_{\mathrm{ijk}}+\left(1-{\hat{\pi }}_{\mathrm{ijk}}\right){\left(\frac{{\hat{\theta }}_{\mathrm{ijk}}}{{\hat{\theta }}_{\mathrm{ijk}}+{\hat{\mu^{\prime }}}_{\mathrm{ijk}}}\right)}^{{\hat{\theta }}_{\mathrm{ijk}}}}{{\hat{\pi }}_{\mathrm{ijk}}\delta_{0}\left(y_{\mathrm{ijk}}\right)+\left(1-{\hat{\pi }}_{\mathrm{ijk}}\right)f_{\mathrm{NB}}\left(y_{\mathrm{ijk}}|{\hat{\mu^{\prime }}}_{\mathrm{ijk}},\;{\hat{\theta }}_{\mathrm{ijk}}\right)}$$

***A. M*-step:** Maximize $Q^{c+1}$ to update the parameter estimates. (i). The parameters from the count component of the model, $\left\{{\hat{\mu }}_{j}^{\prime },\;{\hat{\theta }}_{j}\right\}$, are updated within the GLM framework, as given in Eq. (37).

(37) $$\log\mu_{j}^{\prime }=X\gamma_{j}+Rw_{j}+Cs_{j}+O_{\mu }$$

The updated values of the estimates of parameters at (c + 1)^th^ step is obtained by providing the observation wise weights, ${\hat{w}}_{ijk}^{\left(c\right)}$(Eq. (35)) and parameters estimates at *c^th^* step. For this purpose, the *glm.nb* function in MASS R package was executed. (ii). The zero-inflation probability, ${\hat{\pi }}_{ijk}$, is updated with the logistic regression, can be expressed as:

(38) $$logit\left(\pi_{j}\right)=X\beta_{j}+Ru_{j}+Cv_{j}+O_{\pi }$$

The updated value of ${\hat{\pi }}_{ijk}$ at step (c + 1) is obtained by incorporating the observation level weights, ${\hat{w}}_{ijk}^{\left(c\right)}$ (Eq. (35)) and the parameters estimate at *c*^th^ step. For this, *glm*(…, family= ‘binomial’) function in stat R package was executed.

**Fig. 1:**
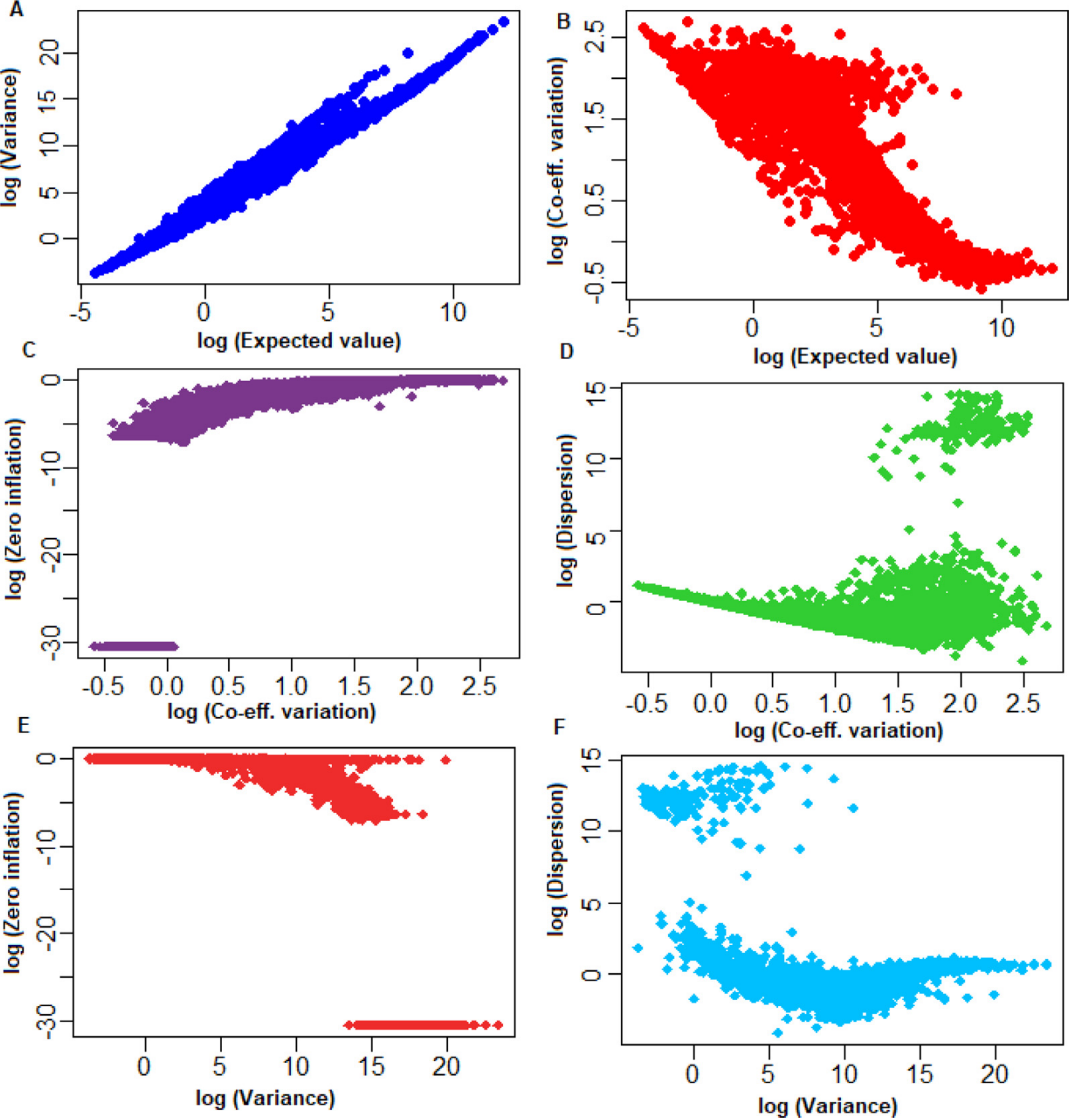
Relationship among the SwarnSeq model parameters with expected value of sample statistics. (A) Expected value *vs.* variance of the observed UMI counts. X-axis: log of the expected value of the observed UMI counts. Y-axis: log of the variance. (B) Expected value *vs.* Co-efficient of variation (CV) of the observed UMI counts. X-axis: log of the expected value of the observed UMI counts. Y-axis: log of CV. (C) Zero-inflation *vs.* CV of the observed UMI counts. X-axis: log of CV. Y-axis: log of zero-inflation. (D) CV *vs.* Dispersion. X-axis: log of the CV. Y-axis: log of Dispersion. (E) Variance *vs.* Zero-inflation observed UMI counts. X-axis: log of the variance. Y-axis: log of zero-inflation. (F) Variance of the observed UMI counts *vs.* Dispersion. X-axis: log of the variance. Y-axis: log of dispersion.

**Fig. 2:**
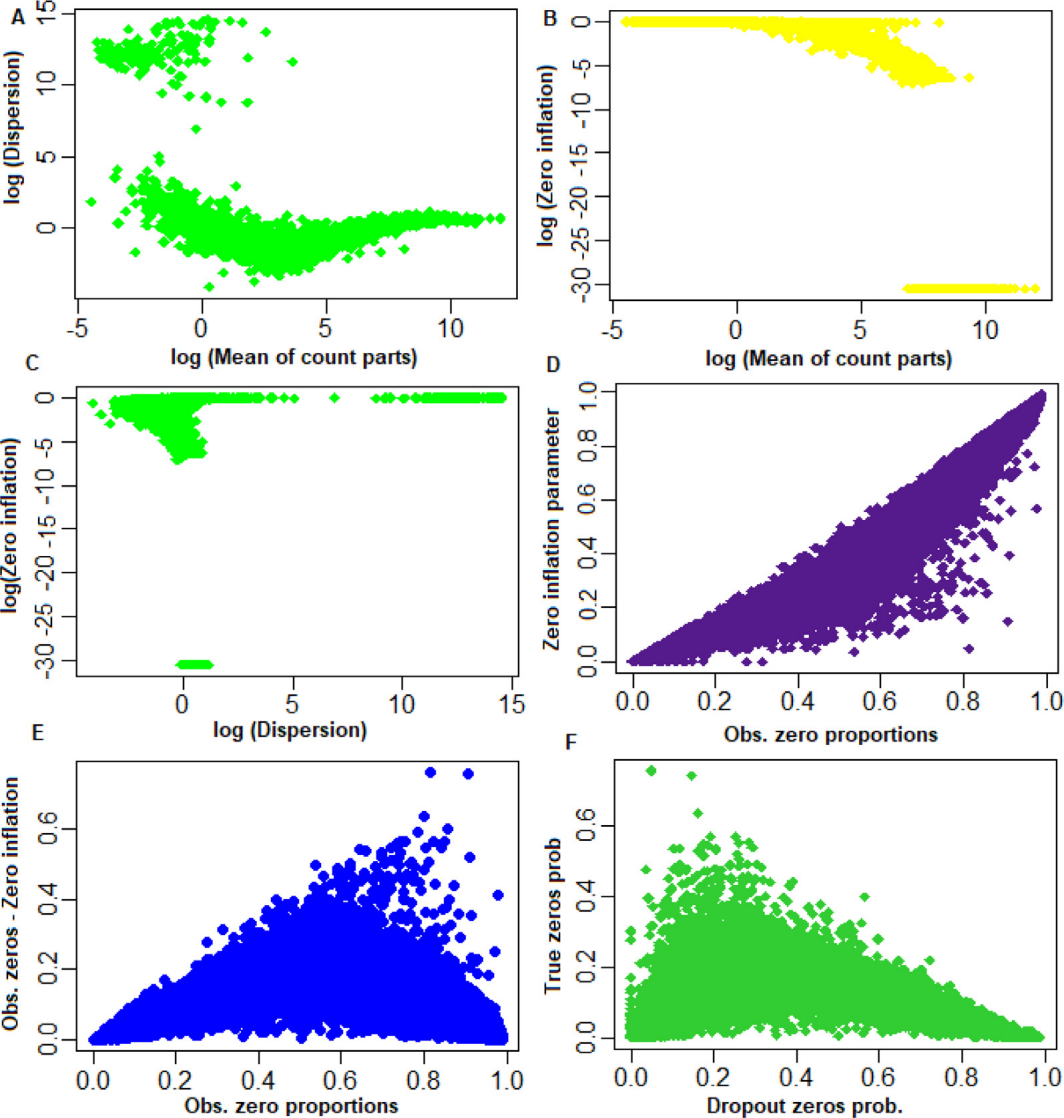
Parameters of the SwarnSeq model estimated through the EM algorithm. (A) Relationship between estimated values of mean with dispersion parameters of genes. X-axis: log of estimated values of means; Y-axis: log of estimated values of dispersions. (B) Relationship between estimated values of mean with zero-inflation parameters. X-axis: log of estimated values of means. Y-axis: log of estimated values of zero-inflation. (C) Relationship between estimated values of zero-inflation with dispersion parameters of genes. X-axis: log of estimated values of dispersion. Y-axis: log of estimated values of zero-inflation. (D) Relationship between estimated values of zero-inflation with observed zero proportions of genes. X-axis: observed means zero proportions. Y-axis: estimated values of zero-inflation parameters. (E) Relationship between observed zero proportions with difference between observed and true proportion of zeros of genes. X-axis: observed means zero proportions. Y-axis: difference between observed and true proportion of zeros. (F) Relation between true and dropout zeros. X-axis: dropout zero probability. Y-axis: true zero probability.

The above procedure is iterated until the convergence is achieved, the detail procedure can be found at [2]. It is important to note that for some genes, the EM algorithm may fail to converge or may be not successful [8]; therefore, we used Nelder's optimization algorithm [6] implemented in *optim* function of stats R package to estimate the MLE of parameters. The developed EM algorithm for estimation of SwarnSeq model parameters was applied to the considered experimental single-cell UMI data. The obtained analytical results are shown in Figs. 1 and 2. Furthermore, relations between the estimated values of parameters for the genes are also shown (Figs. 1, 2).

### Cell capture rate estimation

The distributions of the observed scRNA-seq UMI counts Eq. (10)–((16) and sample statistic(s) including sample mean and variance Eqs. (22)–((25) depend on the value of cell specific capture rate parameter, *p_ijk_*. However, it is extremely difficult to estimate the cell capture rate parameters inside the estimation procedure based on EM algorithm. Hence, one analytical technique is discussed here to estimate the cell capture rate parameters. For computational simplicity, we assume that the cell specific capture rate parameters remain same across all the genes, *i.e.,* $p_{i1k}=p_{i2k}=\ldots =p_{iJk}=p_{ik}$.

*Case 1: External RNA spike-ins data available*

Let, *n* RNA spike-ins are added to each cell's lysate and spike-in transcripts are processed in parallel. This process will result a set of UMI counts for spike-in transcripts. Let, $C_{1},\;C_{2},\ldots ,\;C_{u},\ldots ,C_{n}$ be the respective *m*RNA concentrations of *n* spike-in transcripts added to *i*^th^ (*i=*1, 2, …, *I_k_*) cell of *k*^th^ (*k*=1, 2, …, *K*) cell cluster and let $R_{i1k},\;R_{i2k},\;\ldots ,R_{iuk}\ldots ,\;R_{ink}$ be the observed UMI counts of the *n* spike-in transcripts for *i*^th^ cell, here, $C_{u}\text{and}\;R_{iuk}$ be the molecular concentration and UMI counts of *u*^th^ spike-in transcript. Now, the transcriptional capture rate for *i*^th^ cell in *k*^th^ cell cluster can be estimated through a linear regression equation, given in Eq. (40).

(40) $$R_{iuk}=p_{ik0}+p_{ik}C_{i}+\epsilon_{u}$$

where, $\epsilon_{u}$ is the random error for *u*^th^ spike-in transcript and assumed to follow Gaussian distribution with zero mean and unit variance. Further, ${\hat{p}}_{ik}$, regression co-efficient, is the estimate of the capture rate for *i*^th^ cell in *k*^th^ cell cluster.

*Case 2: RNA spike-ins data not available*

In most of cases, the spike-ins data are not readily available with researchers in single-cell experimental studies. In such situation, the observed cell library sizes [7] can be used to empirically compute the cell specific capture rate. The procedure is given as follows.

Let, $\left(\rho_{1},\;\rho_{2}\right)$ be the range of cell capture rates and $S_{ik}$ be the library size of *i*^th^ cell in *k*^th^ cell cluster and,

(41) $$L_{ik}=log_{10}\left(S_{ik}\right)\;\forall \;i,\;k$$

(42) $${\hat{p}}_{ik}=\rho_{1}+\left(\rho_{2}-\rho_{1}\right)\frac{L_{ik}-L_{min}}{L_{max}-L_{min}}$$

where, $L_{min}$ and $L_{max}$ in Eq. (42) is given in Eq. (43).

(43) $$L_{\min}=\min_{i,k}L_{\mathrm{ik}}\;\text{and}\;L_{\max}=\max_{i,k}L_{\mathrm{ik}}$$

**Fig. 3:**
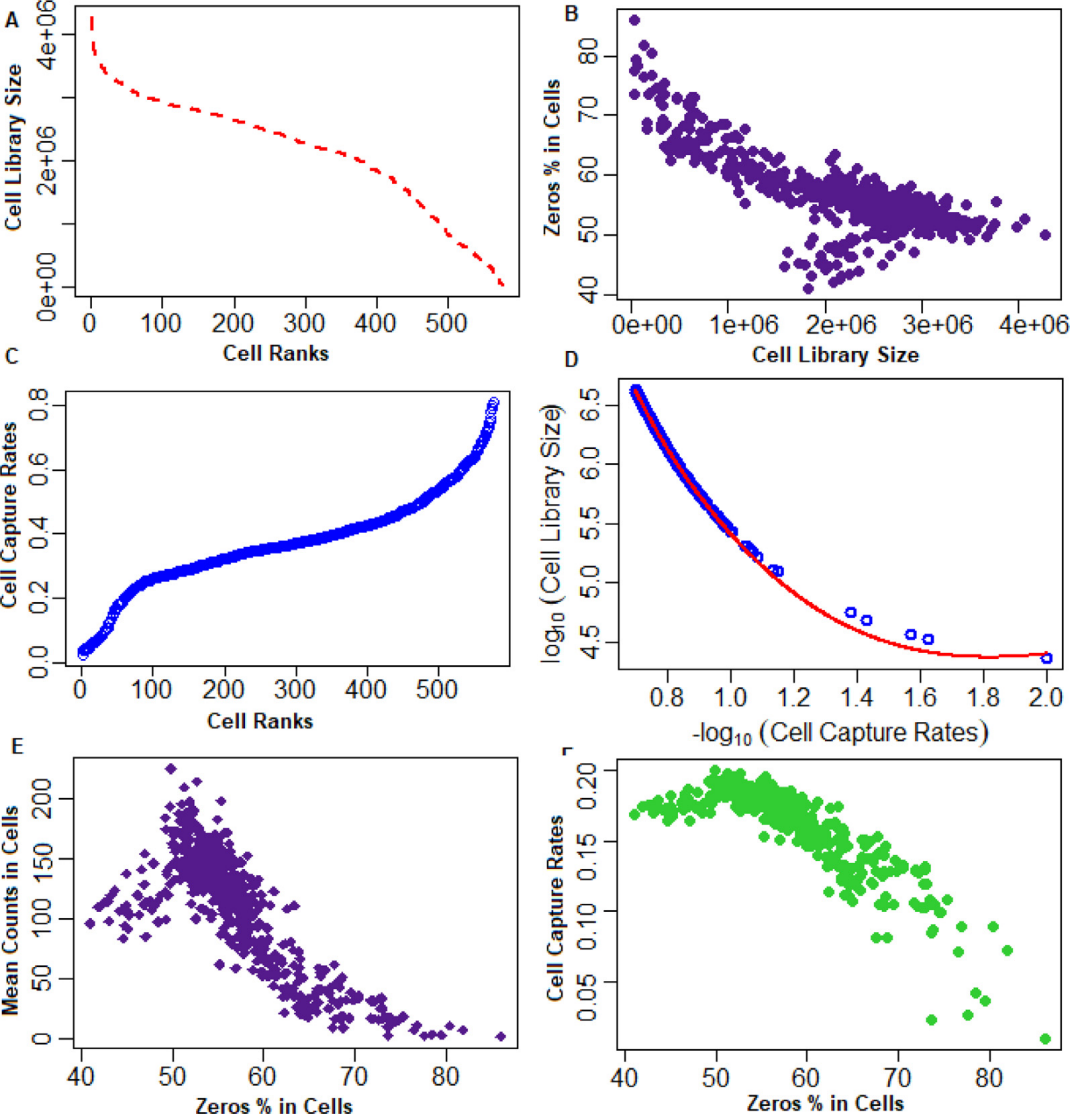
Relationship between the cell specific parameters. (A) Distribution of cell library sizes. X-axis represents the cell ranks; Y-axis represents the cell library sizes. Relationship of cell library sizes with ranks of the cells is s-shaped sigmoid curve. (B) Distribution of cell library sizes with zero counts % in cells. X-axis represents the cell library sizes; Y-axis represents with the zero counts % in cells. Cells with lower library sizes have higher proportions of zero counts as genes expression and *vice-versa*. (C) Relationship of cell capture rates with cell ranks. Here, the cell capture rates are estimated from the external RNA spike-in data. (D) Relationship of cells’ captures rates (estimated from the UMI data) with cell library sizes. The relationship between the capture rates with cell library sizes is bell-shaped. It means the cells with higher library sizes have better cell capture rates and *vice-versa*. (E) Relationship between mean of non-zero counts and zero counts % in cells. X-axis represents the zero counts % in cells; Y-axis represents the mean of non-zero UMI counts. The relation is inversely proportional, *i.e.*, cells with higher zero % have lower mean UMI counts and *vice-versa*. (F) Relationship between capture rates and zero counts % in cells. X-axis represents the zero counts % in cells; Y-axis represents the cell capture rates.

The above procedure for the estimation of cell capture rate parameters was illustrated on the example single-cell dataset and the results are shown in Fig. 3. The estimation of the cell capture rate parameter is shown for the two cases, 1: RNA spike-in data available and 2: RNA spike-in data not available, in Fig. 3.

### Estimated values of parameters from SwarnSeq model

**Fig. 4:**
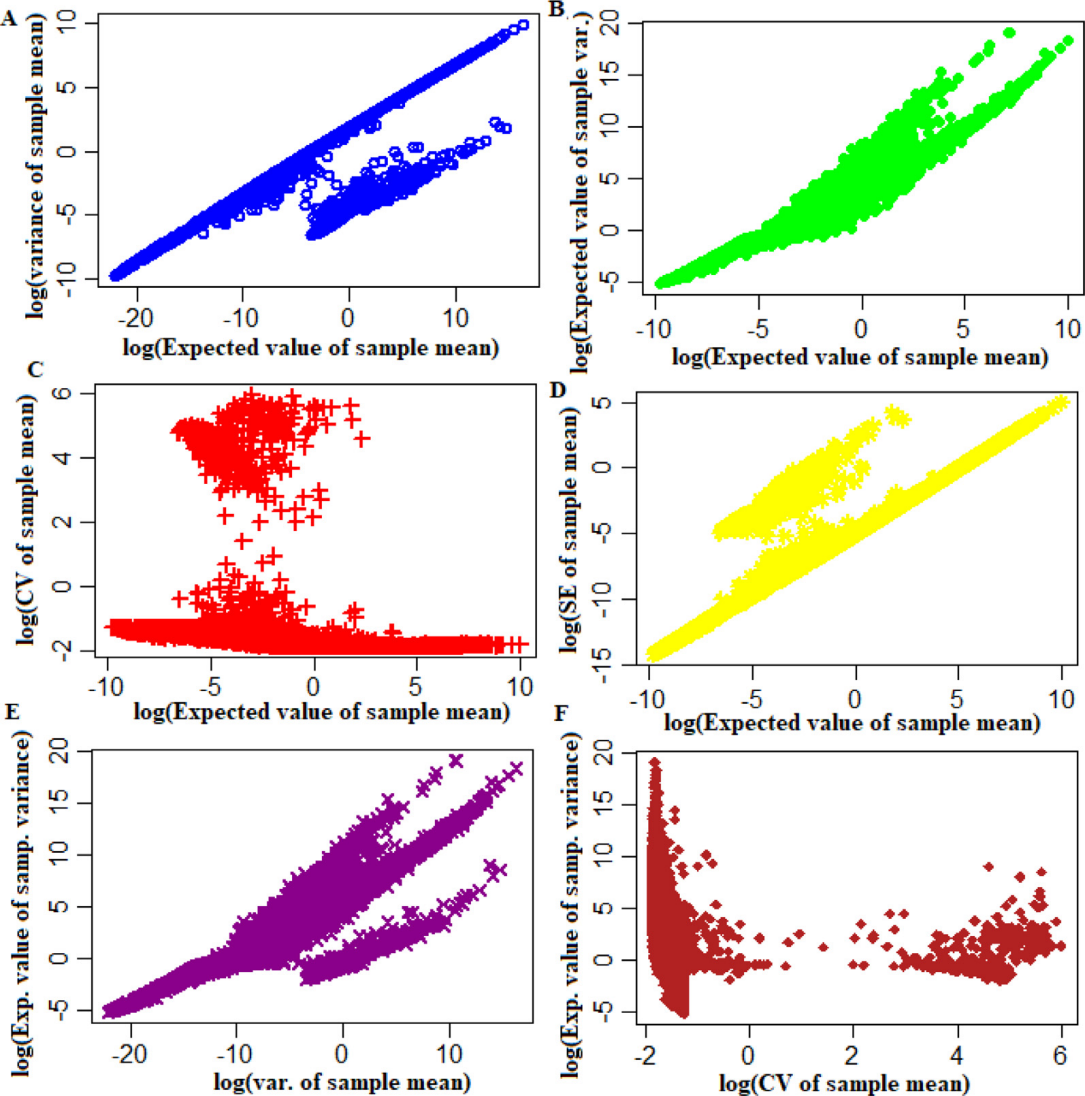
Sample mean and variance of the observed UMI counts of the genes. (A) Expected value *vs.* variance of sample mean plot. X-axis: Expected value of sample mean; Y-axis: Variance of sample mean. (B) Expected value of sample mean *vs.* expected value of sample variance plot. X-axis: Expected value of sample mean; Y-axis: Expected value of sample variance. (C) Expected value of sample mean *vs.* CV of the sample mean plot. X-axis: Expected value of sample mean; Y-axis: CV of sample mean. (D) Expected value of sample mean vs. standard error of sample mean plot. X-axis: Expected value of sample mean; Y-axis: standard error of sample mean. (E) Variance of sample mean *vs.* expected value of sample variance plot. X-axis: Expected value of variance of sample mean; Y-axis: Expected value of sample variance. (F) CV of sample mean vs. expected value of sample variance. X-axis: CV of sample mean; Y-axis: Expected value of sample variance.

Let, $\left({\hat{\pi }}_{j},{\hat{\theta }}_{j},{\hat{\mu }}_{j}\right)$ be the MLE estimates of the parameters for *j*^th^ gene estimated through the EM algorithm and ${\hat{p}}_{ik}$ be the estimate of the cell capture rate for *i*^th^ cell, $\bar{\hat{p}}$ be the average of the cell capture estimates over all the cells. Now, the estimated values of different statistic(s) including expected value of sample mean, sample variance, standard error and co-efficient variation for *j*^th^ gene can be obtained as in Eqs. (44)–(48). Further, these developed formulae was applied to the considered experimental single-cell data, to estimate the distribution of sample means of genes and the results are shown in Fig. 4.

The expression for the estimated value of sample mean is given in Eq. (44).

(44) $$E\left({\bar{y}}_{j}\right)={\hat{\mu }}_{j}\left(1-{\hat{\pi }}_{j}\right)\bar{\hat{p}}$$

The expression for estimated value of variance of the sample mean for *j*^th^ gene can be given in Eq. (45).

(45) $$\hat{V}\left({\bar{y}}_{j}\right)=\frac{{\hat{\mu }}_{j}\left(1-{\hat{\pi }}_{j}\right)}{I}\left(2\bar{\hat{p}}+{\hat{\mu }}_{j}{\hat{\varphi }}_{j}\bar{{\hat{p}}^{2}}\right)+{\left(1-{\hat{\pi }}_{j}\right)}^{2}{{\hat{\mu }}_{j}}^{2}var\left(\hat{p}\right)$$

The expression for the estimate of the expected value of sample variance of *j*^th^ gene is shown in Eq. (46).

(46) $$E\left(s_{j}^{2}\right)={\hat{\mu }}_{j}\bar{\hat{p}}+\frac{{{\hat{\mu }}_{j}}^{2}}{{\hat{\theta }}_{j}}\bar{{\hat{p}}^{2}}+{{\hat{\mu }}_{j}}^{2}\mathrm{var}\left(\hat{p}\right)$$

The estimated value of co-efficient of variation for the sample mean of *j*^th^ gene is expressed in Eq. (47).

(47) $$\hat{CV}\left({\bar{y}}_{j}\right)=\frac{\hat{sd}\left({\bar{y}}_{j}\right)}{\hat{E}\left({\bar{y}}_{j}\right)}$$

where, $\hat{sd}\left({\bar{y}}_{j}\right)=+\sqrt{\hat{V}\left({\bar{y}}_{j}\right)}$

The estimated value of standard error (SE) of the sample mean for *j*^th^ gene can be expressed in Eq. (48).

(48) $$\hat{SE}\left({\bar{y}}_{j}\right)=\hat{sd}\left({\bar{y}}_{j}\right)/\sqrt{I}$$

### Determination of optimum number of cell clusters

The major downstream analysis for scRNA-seq data is cluster analysis, extensively used for detecting various cell types [2,3]. For this purpose, *k*-means clustering technique is used and implemented in various single-cell analytic tools. However, not much work has been done to determine the optimum value of number of cell clusters, to which the cells present in the scRNA-seq data, is categorized. Besides, the SwarnSeq model requires cell cluster information to model the observed UMI counts of the genes. Therefore, we reported an algorithm to determine the optimum number of cell clusters that the cells need to be grouped based on the observed UMI count data, which is given as follows.

Let, $Y_{ik}$: mean expression value of *i*^th^ cell in *k*^th^ cell cluster; $Y_{.k}$: mean expression value of *k*^th^ cell cluster, and ${\bar{Y}}_{...}$ be the over-all mean.

Then, Total Sum of Squares (TSS) can be expressed as:

(49) $$TSS=\sum_{k=1}^{K}\sum_{i=1}^{I_{k}}{\left(Y_{ik}-{\bar{Y}}_{\cdot \cdot }\right)}^{2}=\sum_{k=1}^{K}\sum_{i=1}^{I_{k}}{\left(Y_{ik}-{\bar{Y}}_{\cdot \cdot }\right)}^{2}+\sum_{K=1}^{I_{k}}I_{k}{\left({\bar{Y}}_{.k}-{\bar{Y}}_{\cdot \cdot }\right)}^{2}=WSS+BSS$$

where, WSS: Within cluster sum of squares, BSS: Between cluster sum of squares.

Now, the proposed index to decide the optimum number of cell clusters can be expressed in Eq. (50).

(50) $$r_{h}=\frac{WSS}{BSS}$$

where, $r_{h}>0$ is the index value at *h* number of cell clusters.

**Fig. 5:**
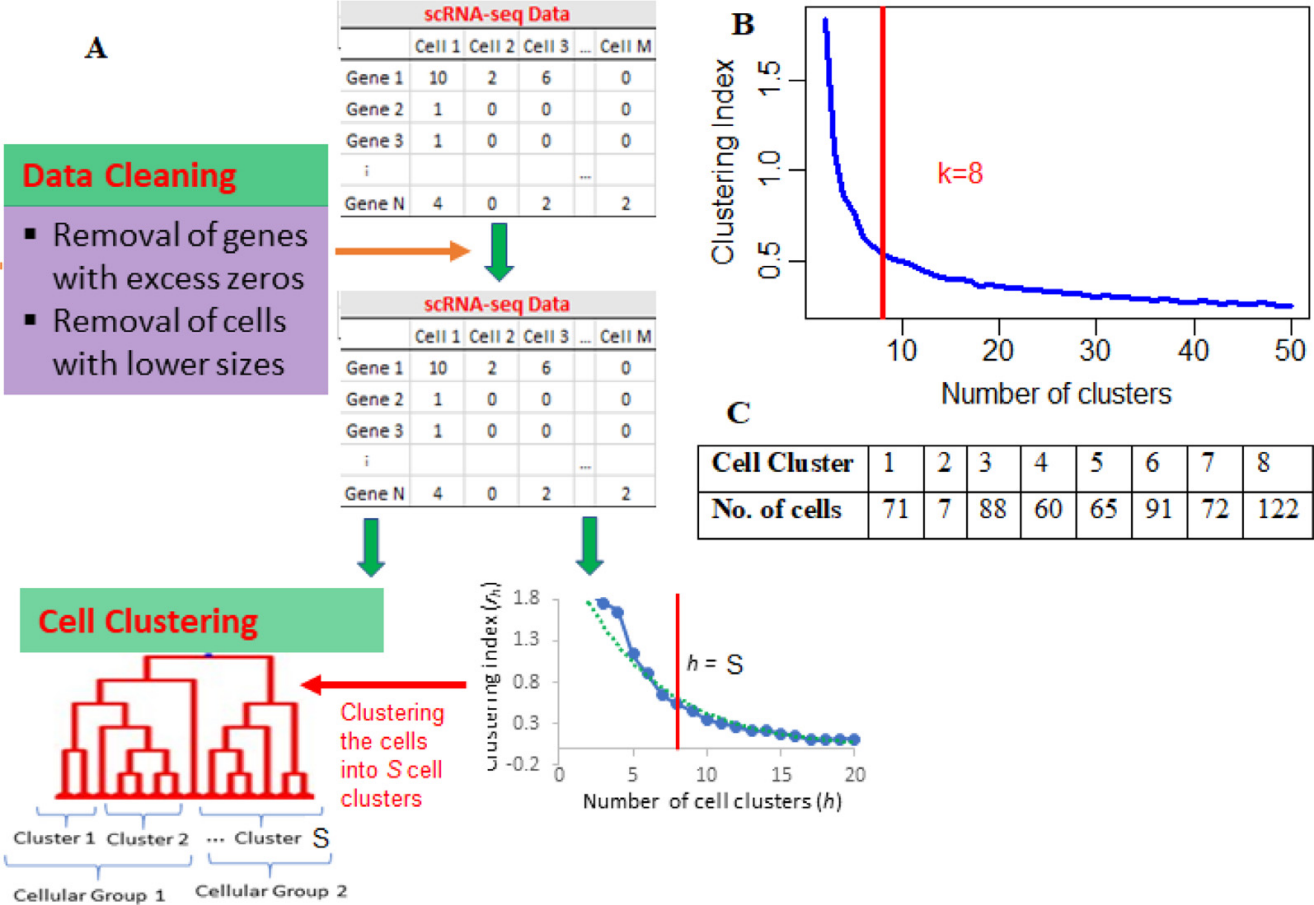
Schematic layout of cluster analysis in SwarnSeq method. (A) Flowchart for cell cluster number determination algorithm. (B) Determination of the optimum number of cell cluster for the experimental single-cell data. X-axis: Number of cell clusters; Y-axis: Clustering indices for every cell cluster. (C) Distribution of the cells across the cell clusters.

In our algorithm, the clustering indices ($r_{h}$) were computed for different values of *h* (≥ 2) using the observed scRNA-seq UMI counts data. Then, the *h* value which provides the maximum value of $r_{h}$can be chosen as the estimator for optimum number of cell clusters for that scRNA-seq data. Alternatively, the optimum value of *h* can be obtained through graphically by plotting *h vs.* $r_{h}$ and choosing the point in x-axis where the curve gets flatten. The algorithm for this reported technique is given in Fig. 5. The algorithm is also implemented in *optimcluster* function of SwarnSeq R package. Further, this algorithm was applied to the considered experimental single-cell data to demonstrate its utility and the results are shown in Fig. 5. For instance, in cluster index *vs.* cluster number plot, the curve has its inflexion point at *k* = 8, means that the 576 cells present in the data can be clustered into eight optimal cell clusters (Fig. 5B). The cluster wise distribution of cells is also shown (Fig. 5C).

### Differential expression analysis of genes

In SwarnSeq approach, the mean parameter of each gene depends on the cellular groups (Eq. (26)). Further, the factors such as cell clusters and cell co-variates are included in the model (Eq. (26)) to remove their unwanted effects on the mean of genes. For DE analysis of genes, two group comparisons are made and the model in Eq. (26) can be expanded as:

(51) $$\log\left(\mu_{ijk}\right)=\gamma_{0j}+\gamma_{1j}x_{ijk}+w_{j1}r_{ij1}+\ldots +w_{jK}r_{ijK}+s_{j1}c_{1ij}+\ldots +s_{Mj}c_{Mij}+O_{\mu_{j}}$$

where, $x_{ijk}$: binary indicator for cellular group membership, $\gamma_{0j}$: (intercept term) logarithm of mean parameter for *j*^th^ gene in the reference cellular group, $\gamma_{1j}$: log Fold Change parameter for *j*^th^ gene, $w_{jk}$: regression co-efficient for *k*^th^ cell cluster for *j*^th^ gene, $r_{ijk}$: indicator variable for cell cluster membership of *i*^th^ cell in *k*^th^ cluster for *j*^th^ gene, $s_{jm}$: regression co-efficient for *m*^th^ (*m* = 1, 2, …, *M*) cell co-variates of *j*^th^ gene, $c_{mij}$: indicator variable for *m*^th^ co-variate of *i*^th^ cell for *j*^th^ gene and $O_{\mu_{j}}$: offset term.

To statistically test whether *j*^th^ gene is expressed differentially or not across the cellular groups, the following hypotheses are tested.

$$H_{0}:\gamma_{1j}=0\;vs.\;H_{1}:\;\gamma_{1j}\neq 0$$

**Fig. 6:**
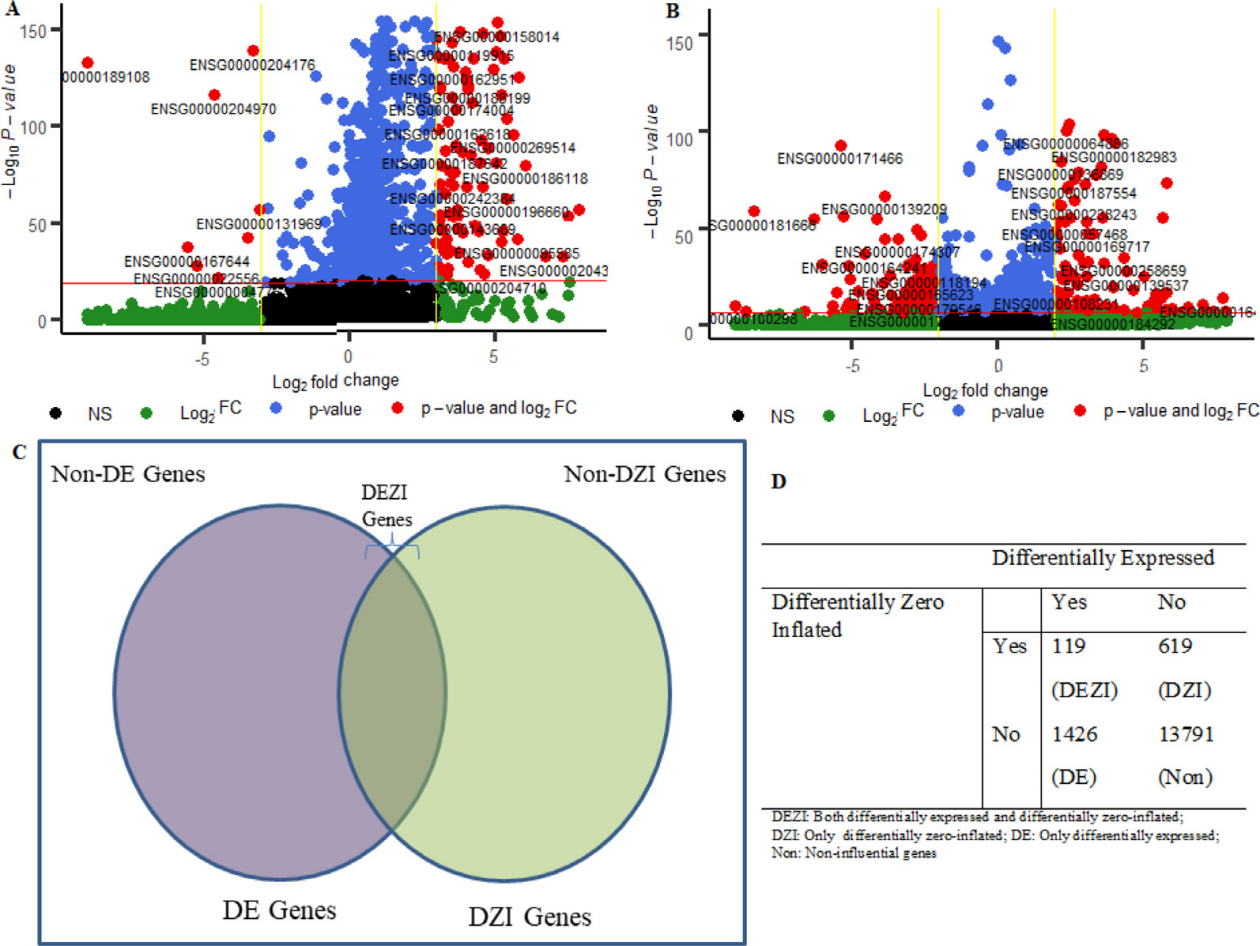
Key analytical results obtained through SwarnSeq Model. (A) Volcano plot for differential expression analysis results. X-axis represents the *log_2_* transformation of the fold change values of genes. Y-axis represents the -*log_10_* transformation of the *p-values* computed through the SwarnSeq model. red color represent the genes whose both -*log_10_ p-values* > 20 and |log_2_FC| > 3; blue color represent the genes whose -*log_10_ p-values* > 20; green color represent the genes whose |log_2_FC| > 3; black color indicates the non-significant genes. (B) Volcano plot for differential zero-inflation analysis results. X-axis represents the *log_2_* transformation of the fold change values of genes. Y-axis represents the -*log_10_* transformation of the *p-values* computed through the SwarnSeq model. red color represent the genes whose both -*log_10_ p-values* > 7 and |log_2_FC| > 2; blue color represent the genes whose -*log_10_ p-values* > 7; green color represent the genes whose |log_2_FC| > 2; black color indicates the non-significant genes. (C) Schematic representation of the classification of key genes detected through SwarnSeq model. DE genes: Differentially expressed; DZI: Differentially zero-inflated; DEZI: Both differentially expressed and differentially zero-inflated; Non-DE: non-differentially expressed; non-DZI: non-differentially zero-inflated. (D) Illustration of SwarnSeq method for classification of influential genes. Numbers in cells represent the genes belong to each category; *(.)*: classes of the genes.

The above test can be performed by using Likelihood Ratio Test (LRT) statistic, and can be expressed in Eq. (52).

(52) $$DS_{j}=-2\left\{l\left(\Omega_{j}={\hat{\Omega }}_{j0}\right)-\;l\left(\Omega_{j}={\hat{\Omega }}_{j}\right)\right\}$$

where, $DS_{j}$: LRT statistic of *j*^th^ gene; ${\hat{\Omega }}_{j0}$: MLE of $\Omega_{j}$ for *j*^th^ gene under the constraint of *H_0_*; and ${\hat{\Omega }}_{j}$: unconstrained MLE of $\Omega_{j}$ for *j*^th^ gene. The test statistic, $DS_{j}$, follows a Chi-square distribution with 1 degree of freedom (for 2 groups) under *H_0_*. Further, based on the distribution of $DS_{j}$, the *p-value* for *j*^th^ gene was computed and this procedure was repeated for all the genes. Then the adjusted *p-values* and FDRs for the genes were computed after adjustment for multiple hypothesis testing. The above statistical methods of DE analysis was illustrated on the considered single-cell dataset [1] and the results are shown in Fig. 6. The volcano plot of the genes obtained through DE analysis is shown in Fig. 6A. The DE analysis results indicated that 274 genes were identified as differentially expressed between the NA19101 and NA19239 cell groups (Fig. 6A) for the considered data.

### Differential zero inflation analysis of genes

In literature, it is well established that the genes in scRNA-seq data are highly zero inflated (*i.e.*, biological and dropout zeros) due to the nature of single-cell studies and several technical, and biological factors [2], [3], [4], [5]. Therefore, it is important to identify the genes which have different number of zeros as expression across the two cellular groups. For this purpose, the SwarnSeq method can perform the zero inflation analysis of the genes across the two cell groups and detect those genes for further study. In SwarnSeq model, the zero inflation parameters of genes depend on the cellular groups through the model given in Eq. (27). Further, factors such as cell clusters and other cell-level auxiliaries are included in the model to remove the unwanted confounded effects from the zero-inflation probabilities of genes. For Differential Zero Inflation (DZI) analysis of genes, two cell groups’ comparisons are made and the model in Eq. (27) can be written as:

(53) $$logit\left(\pi_{ijk}\right)=\beta_{0j}+\beta_{1j}x_{ijk}+u_{j1}r_{ij1}+\ldots +u_{jK}r_{ijK}+v_{1j}c_{1ij}+\ldots +v_{Mj}c_{Mij}+O_{\pi_{j}}$$

where, $x_{ijk}$: binary indicator for cellular group membership, $\beta_{0j}$: intercept term for *j*^th^ gene (reference cellular group), $\beta_{1j}$ is the log Fold Change (zero inflation) parameter for *j*^th^ gene, $u_{jk}$: regression co-efficient of *k*^th^ cell cluster for *j*^th^ gene, $r_{ijk}$: indicator variable for cell cluster membership of *i*^th^ cell in *k*^th^ cluster for *j*^th^ gene, $v_{mj}$: regression co-efficient for *m*^th^ (*m* = 1, 2, …, *M*) cell co-variates of *j*^th^ gene, $c_{mij}$: indicator variable for *m*^th^ co-variate of *i*^th^ cell for *j*^th^ gene and $O_{\pi_{j}}$: offset term.

Statistically to decide whether *j*^th^ gene is DZI or not, the following hypotheses are tested.

$$H_{10}:\beta_{1j}=0\;vs.\;H_{1}:\;\beta_{1j}\neq 0$$

The above test can be performed by using LRT statistic, and its expression is given in Eq. (54).

(54) $$DZ_{j}=-2\left\{l\left(\Omega_{j}={\hat{\Omega }}_{j0}\right)-\;l\left(\Omega_{j}={\hat{\Omega }}_{j}\right)\right\}$$

where, $DZ_{j}$: DZI LRT statistic for *j*^th^ gene; ${\hat{\Omega }}_{j0}$: MLE of $\Omega_{j}$ under the constraint of $\beta_{1j}=0\;$ and ${\hat{\Omega }}_{j}$: unconstrained MLE of $\Omega_{j}$. Here $DZ_{j}$, for all *j*, has a Chi-square distribution with 1 degree of freedom (for 2 groups comparison) under *H_0_*. The adjusted *p-values* and FDR for the DZI analysis were computed for all the genes after adjusting for multiple hypothesis testing through the SwarnSeq method. The above statistical methods of DZI analysis was illustrated on the considered Tung's scRNA-seq data [1]. The volcano plot of the genes obtained through the developed DZI analysis is shown in Fig. 6B. The results indicated that 243 genes were identified as differentially zero-inflated between the NA19101 and NA19239 cell groups (Fig. 6B). In other words, 243 genes have significant number of expressions as zero counts across the NA19101 and NA19239 cell groups.

### Classification of detected influential genes

DE and DZI analyses are two major downstream analytical procedures usually practiced in single-cell experimental studies. Hence, it is interesting to know the group of genes which are expressed differentially across the cellular groups as well as differentially zero inflated. For this purpose, SwarnSeq method is able to classify the detected influential genes into different classes based on DE and DZI analyses, as shown in Fig. 6. For instance, $H_{0}:\gamma_{1j}=0$ detects all the genes, which are expressed differentially, while $H_{10}:\beta_{1j}=0$ detects the genes differentially zero inflated across the cellular groups. Further, the SwarnSeq detects a class of genes in scRNA-seq data with both *H_0_* and *H_10_* rejected. This indicates there is a significant difference in the number of cells with zero values as expression of genes across the cellular groups, but the (non-zero) expressions in the remaining cells show significant differences. This group of influential genes is termed as ‘DEZI’ genes (Fig. 6). The other class of genes, for which *H_0_* is rejected, but *H_10_* is not rejected. This means the class of genes for which there is no significant difference in the number of cells whose expressions are zeros across the cellular groups, but they are expressed differentially. We call this group of genes as only ‘DE’ class genes (Fig. 6). Further, the third type (*i.e.,* only DZI) of genes, for which *H_10_* is rejected, but *H_0_* is not rejected (Fig. 6). It includes the genes for which, there is a significant difference in the number of cells with zero expression values across the two cellular groups, but the (non-zero) expressions in the remaining cells show no significant difference. The utility of the SwarnSeq method for classification of the detected influential genes in scRNA-seq study was demonstrated on one real single-cell data and the results are shown in Fig. 6.

## Conclusion

Statistical analysis of single-cell data in presence of biological confounding factors (leading to severe dropout events) is a challenging task. Therefore in this paper, statistical techniques, implemented in the SwarnSeq, are presented for various analyses of single-cell experimental datasets. The analytical techniques include model fitting, EM algorithm based model parameters estimation procedure, estimation of cell capture parameters, clustering and determination of optimal cell clusters, distribution of observed UMI counts of genes, distribution of sample mean and variance of genes, differential expression, and differential zero inflation analyses, classification of genes, *etc*. A practical real data example was given for illustration of all the analytical techniques in the SwarnSeq. The SwarnSeq method will surely help the experimental biologist and genome researchers to perform various analyses on a single platform. In future, improved parameter estimation procedure including Bayesian techniques can be implemented in the SwarnSeq tool to estimate the gene specific dispersion, and that will enhance its performance. The SwarnSeq method assumes the factors, such as cellular groups, cell clusters and other co-variates, have fixed effects on means and zero inflations. This assumption may not hold good for single-cell data, as some biological factors may have random effects. Therefore, random or mixed effect models can be implemented in SwarnSeq method to improve its performance. The proposed approach is shown with one application in single-cell data analytics and it can be applied in other analytical fields where the data is zero-inflated and over dispersed such as pest population, sample surveys, *etc*. studies.

## Submission type

Direct submission

## CRediT authorship contribution statement

**Samarendra Das:** Conceptualization, Investigation, Data curation, Formal analysis, Methodology, Software, Validation, Visualization, Writing – original draft, Writing – review & editing. **Shesh N. Rai:** Project administration, Supervision, Funding acquisition, Writing – review & editing.

## Declaration of Competing Interest

Authors declare that they have no competing interests.
